# A Possible Mechanism of Graphene Oxide to Enhance Thermostability of D-Psicose 3-Epimerase Revealed by Molecular Dynamics Simulations

**DOI:** 10.3390/ijms221910813

**Published:** 2021-10-06

**Authors:** Congcong Li, Zhongkui Lu, Min Wang, Siao Chen, Lu Han, Weiwei Han

**Affiliations:** Key Laboratory for Molecular Enzymology and Engineering of Ministry of Education, School of Life Science, Jilin University, 2699 Qianjin Street, Changchun 130012, China; congcong17@mails.jlu.edu.cn (C.L.); luzk1619@mails.jlu.edu.cn (Z.L.); minwang20@mails.jlu.edu.cn (M.W.); sachen20@mails.jlu.edu.cn (S.C.)

**Keywords:** graphene oxide (GO), molecular dynamics simulations, thermostability, D-psicose 3-epimerase

## Abstract

Thermal stability is a limiting factor for effective application of D-psicose 3-epimerase (DPEase) enzyme. Recently, it was reported that the thermal stability of DPEase was improved by immobilizing enzymes on graphene oxide (GO) nanoparticles. However, the detailed mechanism is not known. In this study, we investigated interaction details between GO and DPEase by performing molecular dynamics (MD) simulations. The results indicated that the domain (K248 to D268) of DPEase was an important anchor for immobilizing DPEase on GO surface. Moreover, the strong interactions between DPEase and GO can prevent loop α1′-α1 and β4-α4 of DPEase from the drastic fluctuation. Since these two loops contained active site residues, the geometry of the active pocket of the enzyme remained stable at high temperature after the DPEase was immobilized by GO, which facilitated efficient catalytic activity of the enzyme. Our research provided a detailed mechanism for the interaction between GO and DPEase at the nano–biology interface.

## 1. Introduction

In the past several decades, enzymes have been widely applied in industrial production, biopharmaceutical research, and medical diagnosis, due to its reproducibility and ability for increasing the reaction rate without changing the equilibrium [1,2]. However, the use of enzymes in industrial production has still had some limitations, such as operation stability, temperature, susceptibility to reaction condition, etc. [3]. In particular, high temperatures may result in structural denaturation of enzymes, leading to loss of catalytic performance of biological catalysts [4]. To address these difficulties, enzyme immobilization technology has been developed to improve thermal stability of enzymes in the wake of the development of nanoparticles [3,5]. At present, it has been reported that a variety of nanoparticles can successfully improve the thermal stability of enzymes [6,7,8,9].

Traditional surface analysis tools are commonly used to analyze the surface characteristics of nanomaterials, but it is generally difficult to capture the dynamic changes between GO and DPEase. However, MD simulation method could overcome this bottleneck. The combination of the two methods can more overall understand the characteristics of the nano−bio interface, providing favorable help for the study of the functions of enzymes [10,11,12,13].

Graphene oxide (GO) is a kind of nanomaterial that has attracted extensive attention in recent years [14,15,16,17]. It is a unique two-dimensional carbon network structure, which has some special properties, such as large surface area [18], extraordinary mechanic stability [19], and good biologic compatibility [20]. It can be used as an immobilization material without coupling reagents or surface modification due to the existence of oxidative functional groups [16,21]. Several enzymes have been successfully immobilized on GO with robust immobilization support by GO nanosheet [22,23,24,25,26].

**Figure 1:**
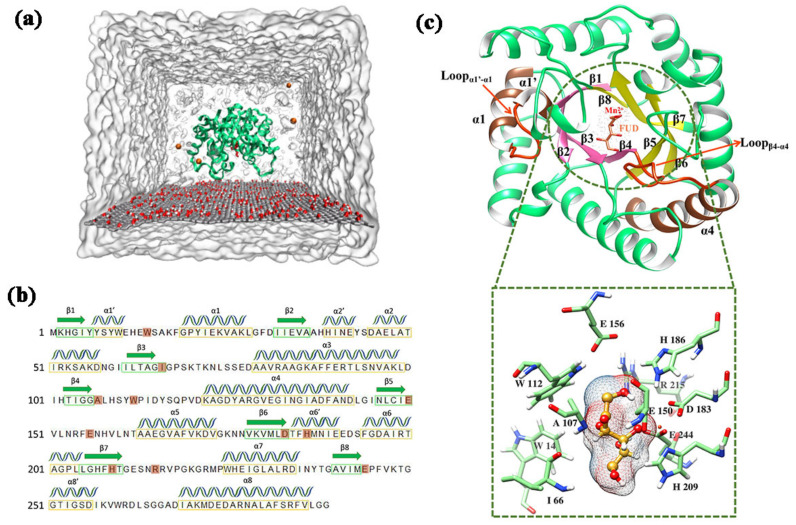
General overview of the initial structure. (**a**) MD simulation box of GO and DPEase-FUD complex with water (the orientation of complex is randomly selected); (**b**) secondary structural elements of DPEase, the helices, and β-strands are displayed in different symbols. Active site residues highlighted in orange; (**c**) molecular architecture of DPEase-FUD and the detailed close-up of the residues interacting with FUD.

Recently, interactions between GO and enzymes have attracted researchers’ attention [27,28]. The recent study revealed that the thermal stability of *Agrobacterium tumefaciens* (*agtu*) DPEase was enhanced by immobilizing enzymes on GO nanoparticles. The *agtu*-DPEase is most active at 50 °C, while the *agtu*-DPEase immobilized by GO shows maximum activity at 60 °C [29]. However, the mechanism of the DPEase adsorption on GO at atomic level is still unclear. In this study, four 300ns time-span MD simulations (Figure 1a) were carried out to explore the interaction between GO and DPEase. D-Psicose is a kind of sugar beneficial for good health but was rare found in nature [30,31]. It is difficult to synthesize by chemical methods [32,33]. DPEase can be used to produce D-Psicose from D-Fructose (FUD) [34]. The secondary structure of the enzyme was shown in Figure 1b. Figure 1c displayed the detailed structure features and active site residues of the enzyme, the important regions have been highlighted. Our research not only revealed the interaction mechanism of GO with DPEase but also provided clues for the design of mutant DPEase to improve the thermal stability of enzyme.

## 2. Results

### 2.1. Structural Stability and Flexibility Analysis

To explore the effect of GO on the thermal stability of DPEase, four models were constructed in the study. Model 1: DPEase complexed with the FUD was simulated at 50 °C. Model 2: the initial structure was the same as Model 1, but was performed at 60 °C. Model 3: the DPEase-FUD with randomly selected direction was placed above GO and was simulated at 50 °C. Model 4: The initial structure was the same as Model 3, but was carried out at 60 °C.

**Figure 2:**
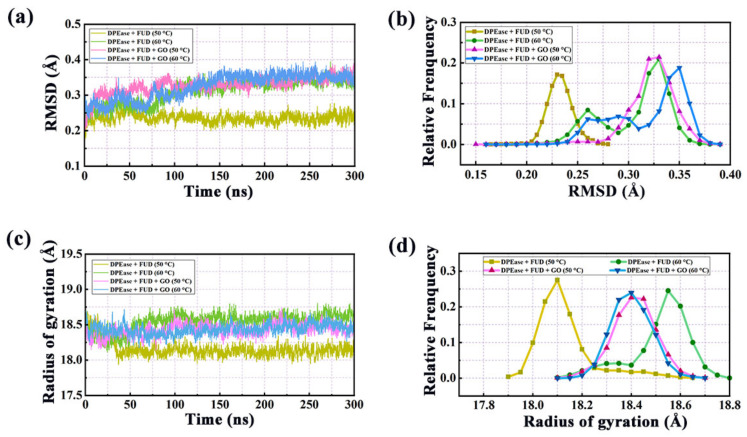
(**a**) Time evolution of RMSDs for backbone atoms in four systems throughout the 300 ns MD simulations; (**b**) corresponding frequency for RMSD; (**c**) radius of gyration changes in four systems during simulations; (**d**) relative frequency for radius of gyration.

For the qualitative study on the stability and convergence of the simulated systems, the root mean square deviations (RMSD) of the protein backbone atoms in regard to the original structure were calculated and the results were shown in Figure 2a. Model 1 maintained a relatively stable RMSD fluctuation during the entire 300 ns of simulations, especially after 75 ns. However, when DPEase-FUD was simulated at higher temperature (Model 2) or were adsorbed on GO (Models 3 and 4), relatively large structural changes were observed. These three systems all reached equilibrium state in around 150 ns. Models 3 and 4 took longer to reach equilibrium, because they underwent the adsorption process on GO. As shown in Figure 2b, RMSD values were mainly distributed in 3.25–3.50 Å, except for Model 1, in which values were mostly concentrated at 2.26 Å.

Radius of gyration (*R_g_*) can provide an insight into the overall size and dimensions of the proteins. *R_g_* was calculated to examine structural drifts in protein complexes for four systems. Figure 2c,d illustrated the calculated results of *R_g_* and corresponding probability for four systems. The *R_g_* values of four systems were stable at 18.10, 18.55, 18.40, and 18.40 Å, respectively. The higher *R_g_* was observed for Model 2, which meant that the protein in Model 2 took place greater conformational change. By contrast, the value of Model 1 was the smallest, indicating that the protein in Model 1 was the most compact during MD simulation. The protein adsorbed on GO (Model 4) presented more coherent than that of the enzyme without the presence of GO (Model 2) under high temperature simulation conditions.

Additionally, to explore the structural divergence of various important parts, the RMSD from different areas, including the loop α1′-α1 (E11-F18), loop β4-α4 (A107-D121), loop β5-α5 (V151-T162), α8′ (G251-D256), and α5 (A163-V173), which have great significance for the catalytic activity of enzyme, were also calculated (Table 1). In the case of the loop β5-α5 and α5, Model 1 exhibited similar trend in deviations with the other three systems. For other regions, Models 2, 3, and 4 presented significantly higher deviations than Model 1. Compared to Models 3 and 4, the average RMSD of the loop α1′-α1 showed larger deviations in Model 2. The average RMSD values for loop β4-α4 and α8′ had similar deviations in Model 2, 3, and 4.

### 2.2. Adsorption of DPEase onto GO

**Figure 3:**
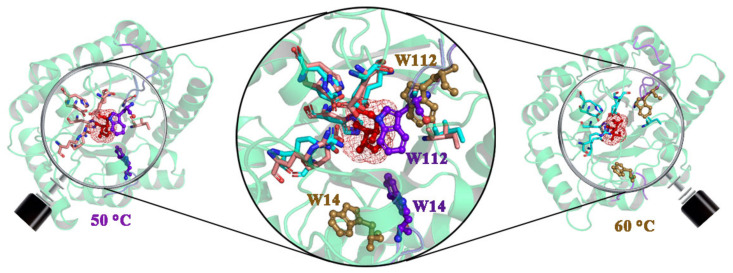
Representative structure for the complex at 50 °C and 60 °C. Additionally, the superimposition of two structures, in which DPEase are displayed in cartoon and active pockets, are represented in different color sticks.

To compare the structural differences between Model 1 and Model 2 during MD simulations under different temperature conditions, the representative structures of the two complexes were plotted in Figure 3.

**Figure 4:**
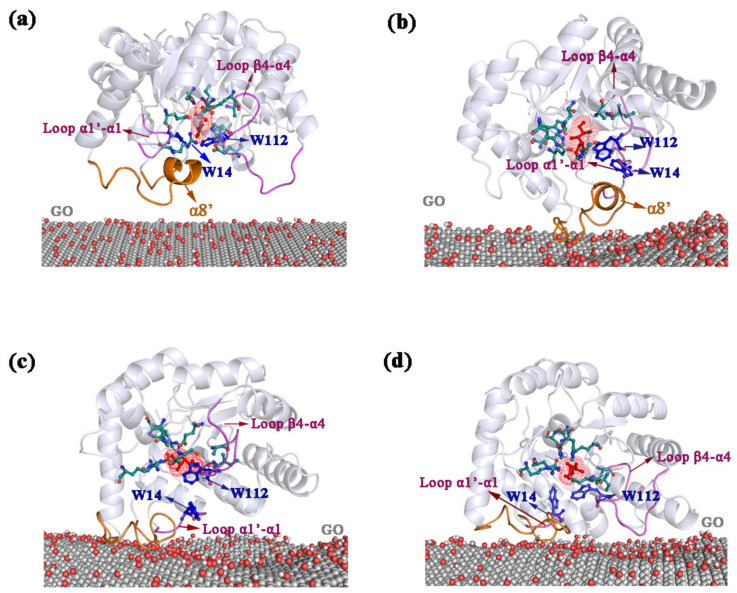
Adsorption process of enzyme on GO at 60 °C. The representative snapshots of (**a**) 0 ns; (**b**) 30 ns; (**c**) 70 ns; and (**d**) 100 ns.

As a result, the two residues (W14 and W112) in active pocket produced distinct alterations in the conformation for two complexes. In contrast, the other residues of active pocket showed minor difference. W14 and W112 were away from the ligand upon high temperature (60 °C) simulation, while at 50 °C, two residues were still tightly bound to the ligand. Since W14 and W112 belong to loop α1′-α1 and loop β4-α4, respectively, the dissimilar displacement of these two residues may be caused by the different dynamic characteristics of loop α1′-α1 and loop β4-α4. According to the above results, it can be speculated that GO could help enhances the thermal stability of enzyme probably by enhancing the stability of these two loops. In order to confirm this hypothesis, we next analyzed the protein adsorption process on GO. Figure 4 showed representative snapshots of the adsorption process for DPEase onto the GO surface under the condition of 60 °C.

As shown in Figure 4a, the initial distance between GO surface and the center of mass (COM) of enzyme that randomly selected orientations was 4.0 nm (Figure 4a). Along the MD simulations, DPEase was gradually adsorbed onto the surface of GO. Firstly, the enzyme moved close to the surface of GO (∼30 ns) through long-range Coulomb interactions between cationic residue of DPEase (R261) and GO (Figure 4b). Secondly, the α8′ and its near loop (from to K248 to D268) served as an anchor (Figure 4c) and was adsorbed onto the surface of GO (70 ns). Finally, the loop β4-α4 was also adsorbed onto the GO surface at 100 ns (Figure 4d), so the protein had larger contact area with GO. The distance between them decreased to ∼0.6 Å. As shown in Figure 4, when the protein was immobilized by GO, although the MD simulation was performed at high temperature, the W14 and W112 were finally tightly bound to the ligand, whereas, in the absence of GO, the two residues were far away from the ligand. Representative conformation for the adsorption process of DPEase on GO surface at 50 °C were displayed in Figure S1, the results were similar to those at 60 °C.

In summary, DPEase can be successfully adsorbed on the surface of GO, resulting in enhancing the stability of the enzyme’s active sites. Table 2 listed the occupancies of formed hydrogen bonds between proteins and ligands in the MD simulations for different systems. Table 2 showed that the hydrogen bond interactions between proteins and ligands significantly were weakened when the temperature increased. While the protein was adsorbed by GO, the hydrogen bond interactions between them were significantly enhanced. The stronger the hydrogen bond interactions, the more stable the interaction between DPEase and FUD. Stable systems will help maintain the catalytic activities of enzymes. So, GO could help improve the stability of DPEase-FUD at high temperature.

**Figure 5:**
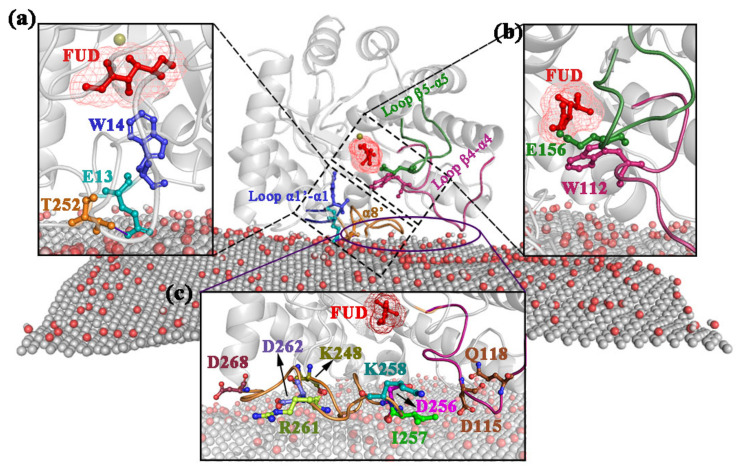
The detailed close-up of the interaction surface between DPEase and GO. (**a**) The interaction between loop α1′-α1 and α8′; (**b**) the interaction between loop β4-α4 and loop β5-α5′; (**c**) structure diagram of the adsorbed region. The key residues have been marked in the picture with different color sticks.

With the purpose of investigating the interaction mechanism of GO with DPEase, the critical residues at the nano–bio interface were discussed in detail. At first, almost all residues (residues K248 to D268) directly interacting with GO were hydrophilic residues, especially lysine and aspartic acid. These residues consisted of K248, S255, D256, K258, W260, R261, D262, S264, D268, D115, and Q118. These key residues had been annotated in Figure 5c. These hydrophilic residues could form strong hydrogen bonds with epoxide and/or carbonyl groups on the surface of GO. Among these residues, K248, K258, and R261 were cationic residues, which can form favorable interactions with anionic GO. Moreover, W260 was aromatic residues, which contributed the π–π stacking and CH–π interactions. Therefore, DPEase was susceptible to be effectively adsorbed onto the GO surface with the assistance of these residues during MD simulations. W252 in α8′ had strong interaction with W13 (Figure 5a), while W112 in loop β4–α4 can form a strong hydrogen bond with E156 (Figure 5b). From Figure 5a,b, we found that W14 and W112 were closed to the adsorbed regions (loop β4–α4, K248 to D268) of the enzyme.

### 2.3. Dynamics of Regions Adsorbed by GO

#### 2.3.1. Dynamic Analysis of Interaction between Loop α1′-α1 and α8′

**Figure 6:**
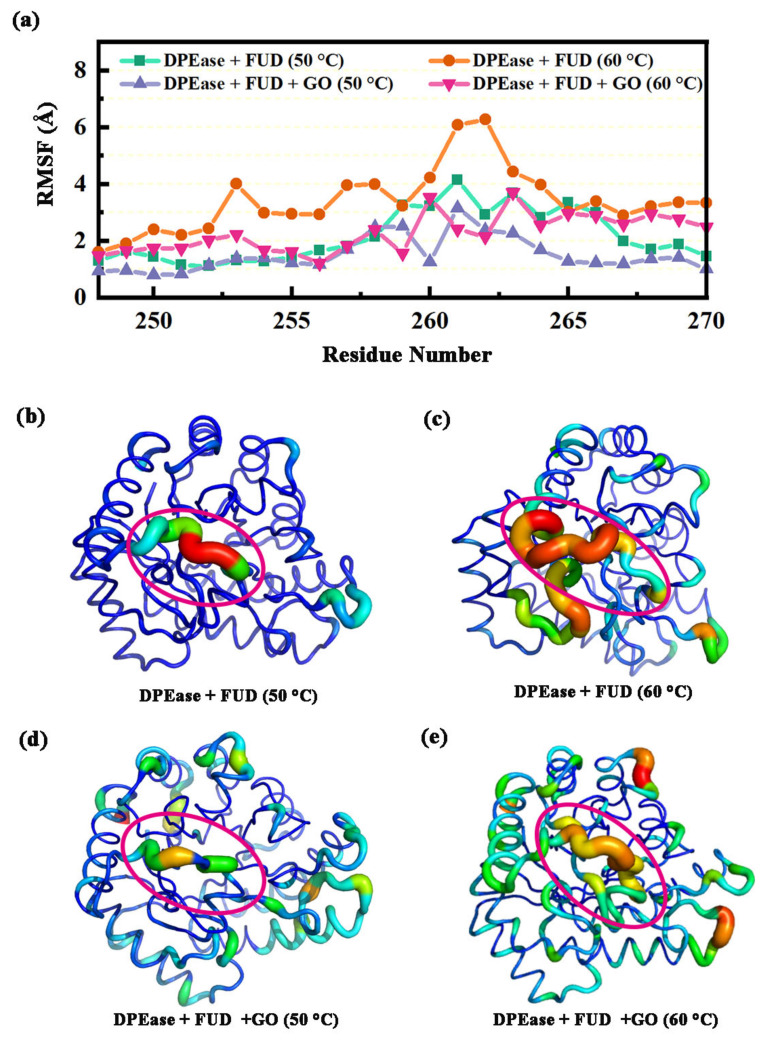
(**a**) The RMSF values of residues 248–268 for four systems; visualizations of the backbone flexibility for (**b**) DPEase + FUD (50 °C); (**c**) DPEase + FUD (60 °C); (**d**) DPEase + FUD + GO (50 °C); (**e**) DPEase + FUD + GO (60 °C).

Previous analysis had identified that K248 to D268 were key residues for protein anchoring on GO. Therefore, we analyzed the dynamic change characteristics of this region. This region includes two parts, α8′and its near loops. The regional fluctuation characteristics can be estimated by computing the root-mean-square fluctuation (RMSF) of Cα atoms for each residue based on MD trajectories. We adopted the equilibrium part of each trajectory to calculate RMSF to ensure the reliability of the results. The calculated RMSF results for this region (K248 to D268) were shown in Figure 6a. It can be seen in Figure 6a that this region in Model 2 was more flexible compared to the other three Models. The reason may be that a high simulated temperature will destroy the stability of the region, resulting in increased atomic fluctuation. Figure 6b–e visually exhibit large fluctuations in Model 2 compared to the other systems. For the complex with GO, whether at 50 °C or 60 °C, atomic volatility was significantly lower than that of Model 2. Based on the above analysis, it can be concluded that this region was successfully adsorbed on GO, resulting in reduced atomic volatility. It can be seen in Figure 6a that R261 and D262 in Model 2 had the largest fluctuation, while the RMSF values decreased significantly in Models 3 and 4, becoming even lower than that in Model 1. This was because R261 and D262 were important residues for protein anchoring on GO, and they can interact strongly with GO. Furthermore, this region contains an α8′. For the RMSF values of this α-helix, those of Model 2 were the largest (2.10–4.00 Å), those of Models 1 and 3 were similar and minimal (0.90–1.50 Å), and those of Model 4 fluctuated between 1.00–2.10 Å.

**Figure 7:**
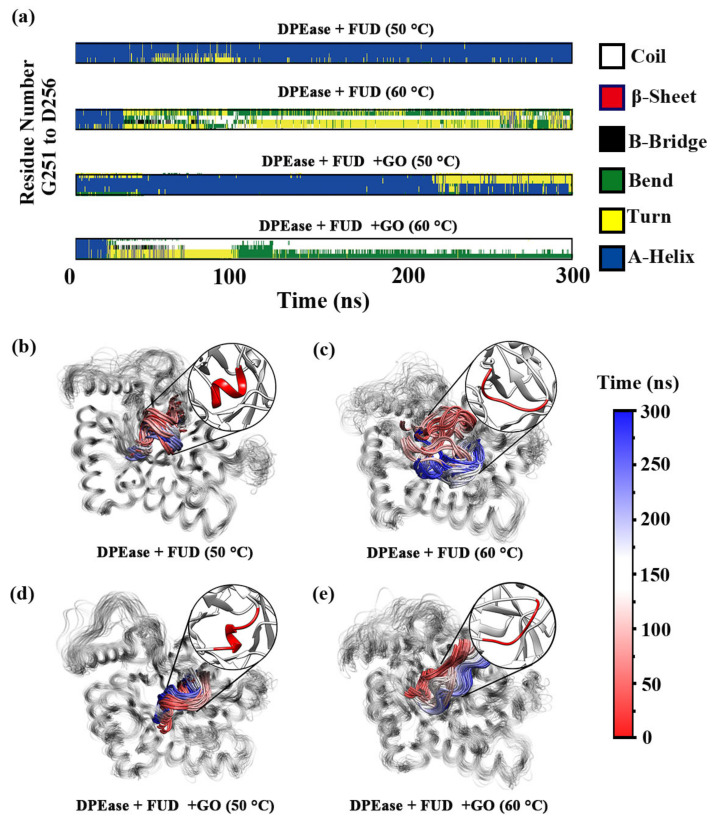
(**a**) DSSP results of α8′ residues; α8′ region is colored mapped from red (0 ns) through white and to blue (300 ns) along with time for (**b**) DPEase + FUD (50 °C); (**c**) DPEase + FUD (60 °C); (**d**) DPEase + FUD + GO (50 °C); (**e**) DPEase + FUD + GO (60 °C).

In order to explain the reasons for this change, we analyzed the time-dependent secondary structure of α8′. Figure 7a presented the detailed changes of secondary structures for the four systems during the 300 ns MD simulations. It was obvious that the helical structure remained stable throughout the simulation process when the complex without GO was simulated at 50 °C, whereas the helix structure developed turns and bends when the simulation was performed at 60 °C. Thus, temperature had an important effect on the stability of helix for DPEase. At 50 °C, the helical structure was essentially maintained. However, after 20 ns, some of the helix became loose, which may be related to the strong adsorption of GO. In contrast, the helix completely coiled and bent after 20 ns at 60 °C.

Figure 7b–e showed overlapping graph of structural changes over time in each system. Model 1 always maintained stable spiral structure with slightly structural change displacement (Figure 7b). Compared with the structures in Figure 7c,d, it can be concluded that, although the simulation at high temperature made the helix uncoiled, the stability of the uncoiled structure was significantly enhanced and the volatility of the structure reduced after the complex was immobilized by GO. For Model 3, even though the helix should remain stable at 50 °C, the helix structure may change slightly due to the strong adsorption of GO (Figure 7d).

**Figure 8:**
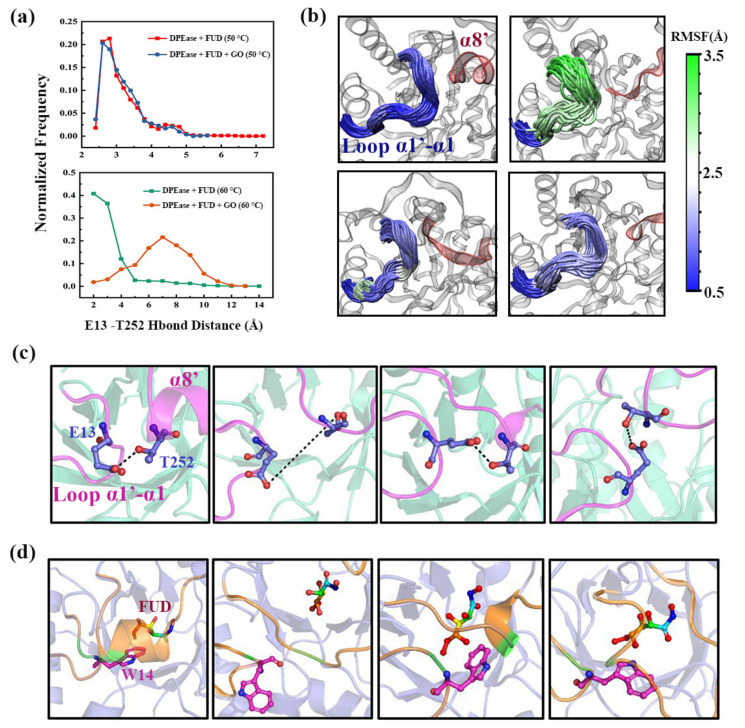
(**a**) Relative frequency of distance distribution for the 300 ns MD simulation; (**b**) loop α1′-α1 residues are colored mapped from green (most flexible) through white to blue (least flexible) according to their calculated Cα RMSF; (**c**) representational structure diagram of the distance between E13 and T252; (**d**) representation snapshots of relative positions between W14 and FUD.

Subsequently, we further analyzed why this region was immobilized on GO to improve the thermal stability and catalytic activity of the enzyme. E13 in loop α1′-α1 can form a strong hydrogen bond interaction with T252, which belongs to α8′. The W14 next to E13 is an active residue that contributed to enzymatic reaction [35]. Therefore, the movement of E13 will affect the dynamic change of E14. We analyzed the variation of hydrogen bond distance between E13 and T252 in the MD simulations. The relative frequency plots for four systems are displayed in Figure 8a. Meanwhile, the representative structure of the distance between the two residues for each system is shown in Figure 8c. The relative position diagram of W14 and ligand is represented in Figure 8d. From Figure 8a, it can be seen that, for Model 1 and 3, the variation trend of distance between E13 and T252 were similar, and the distance between them was mainly distributed about 2.50–2.80 Å, indicating that the stable hydrogen bond can be formed between E13 and T252 during MD simulations. However, the results for Models 2 and 4 were quite different. When the complex was at 60 °C, the distance between the two residues was mainly distributed at about 6.90 Å, so the probability of forming hydrogen bonds was relatively small.

However, when the complex was adsorbed on GO, the distance between E13 and T252 was mainly concentrated in 2.00 Å, which can form stable hydrogen bonds. Loop α1′-α1 is an important structure for enzyme catalysis, in which E13 can form hydrogen bonds with T252, and W13 is an active site residue. The dynamic study for loop α1′-α1 was conducive to a better explanation of the catalytic mechanism. Therefore, RMSF calculation of the loop was performed. The residues contained in loop α1′-α1 were colored mapped from green (most flexible) through white to blue (least flexible), according to their calculated Cα RMSF. Figure 8b showed that the loop α1′-α1 of Model 2 had the largest fluctuation under the influence of high temperature, while the stability of Model 4 was significantly enhanced. Therefore, GO-immobilized enzymes could enhance the stability of loop α1′-α1 through the interaction of hydrogen bonds, thereby helping to improve the stability of the complex.

#### 2.3.2. Dynamic Analysis of Interaction between Loop β4-α4 and β5-α5

From the above analysis for the adsorption process of enzymes on GO (Figure 5b), it can be seen that, with the adsorption process proceeds, the loop β4-α4 also moved close to the surface of GO. It was reported that the loop β4-α4 serves as the lid to the active site [35]. This showed that the importance of the loop β4-α4 on enzyme catalytic. W112 and E156 belong to loop β4-α4 and β5-α5, respectively, and a strong hydrogen bond formed between W112 and E156 during MD simulations. Our analysis of the interaction between loop β4-α4 and loop β5-α5 could help explain the mechanism of thermal stability improvement of GO immobilized enzyme.

**Figure 9:**
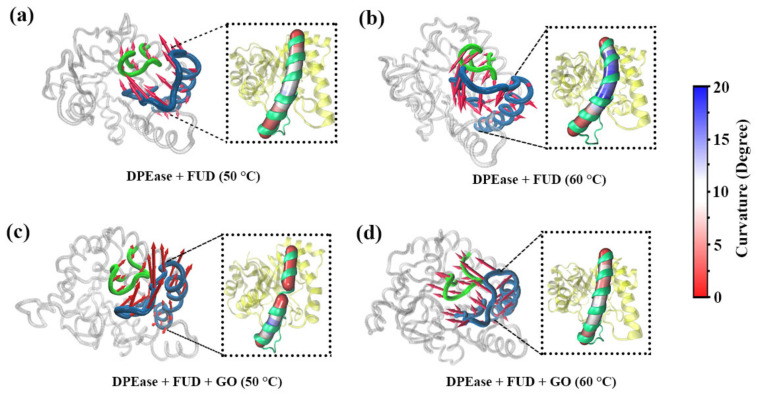
Collective motions along the first eigenvector obtained from principal component analysis for four systems: (**a**) DPEase + FUD (50 °C); (**b**) DPEase + FUD (60 °C); (**c**) DPEase + FUD + GO (50 °C); (**d**) DPEase + FUD + GO (60 °C). Loop β4-α4 is shown in green tube, while loop β5-α5 and α5 are displayed in skybule tube. Red arrows represented movement trends. α5 residues were colored mapped from blue (most curved) through white and to red (least curved) according to their calculated curvature.

To further decode loop β4-α4 and loop β5-α5 conformational changes of DPEase due to the effect of temperature and GO immobilization, the motion directions and magnitudes of the first eigenvector in the four systems were visually displayed using porcupine plots by means of PC analysis (Figure 9).

As shown in Figure 9a, loop β4-α4 and loop β5-α5 in Model 1 represented strong motion consistency, and they all moved toward the catalytic center. Additionally, α5 was consistent with their direction of motion and the degree of curvature was relatively low. In Figure 9b, the complex without GO was carried out at 60 °C, it was obviously that both loop β4-α4 and loop β5-α5 moved away from the active center. Meanwhile, the degree of curvature for α5 was highest in four systems, this may be the reason why the loop moved away from the active center. For Model 3 (Figure 9c), two loops moved away from GO, and they also moved away from the catalytic center. Notably, α5 broke into two parts. As shown in Figure 9d, both loop β4-α4 and loop β5-α5 moved toward the catalytic center and α5 showed smaller curvature than the others.

**Figure 10:**
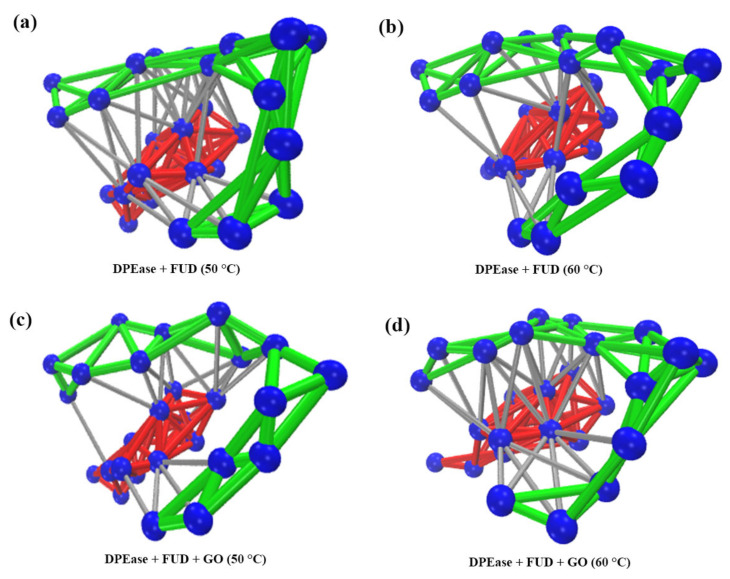
Dynamical network analysis of the interaction between loop β4-α4 and loop β5-α5 in (**a**) DPEase + FUD (50 °C); (**b**) DPEase + FUD (60 °C); (**c**) DPEase + FUD + GO (50 °C); (**d**) DPEase + FUD + GO (60 °C). The nodes (blue) are αC atoms, the red and green edges represent the interactions between αC atoms of the residues for loop β4-α4, loop β5-α5, respectively, and the silver edges show the interactions between αC atoms of two loops.

The dynamical network for interfaces between loop β4-α4 and loop β5-α5 were obtained to describe the stable interaction communities. Figure 10 showed the number of interacting residues in two loops in four systems. More residues participated in the interaction between loop1 and loop2 in Model 1 (Figure 10a) and Model 3 (Figure 10c) than the other two systems (Figure 10b,d). Compared with the Model 1, a weaker interaction between loop1 and loop2 was shown in Model 2 due to the influence of high temperature conditions, so the number of interaction residues was relatively low. The results of comparison between Model 3 and Model 4 showed that only at high temperature, DPEase adsorption on GO resulting in stability of loop β4-α4 be significantly increased, and it was not necessarily good at 50 °C.

**Figure 11:**
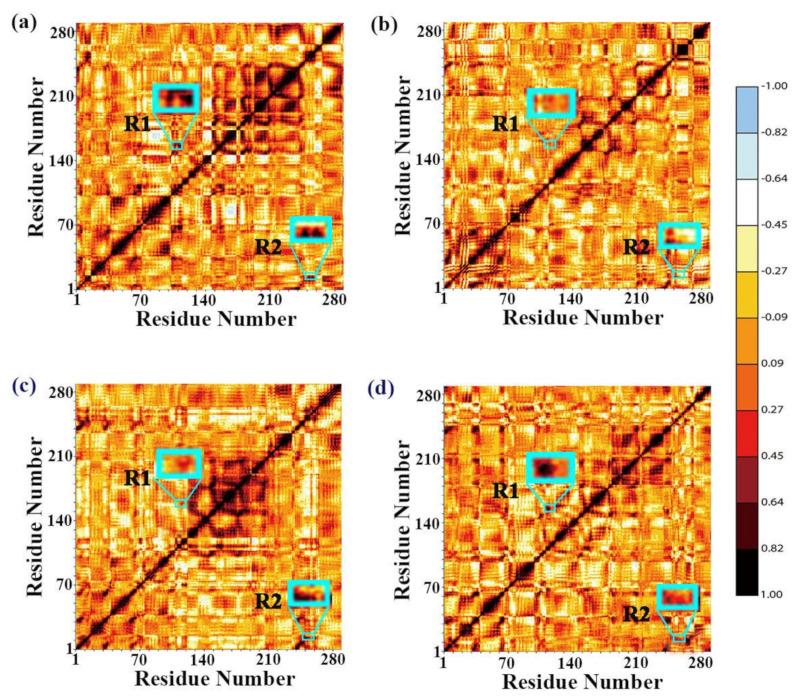
Cross-correlation matrices between residues calculated for four systems: (**a**) DPEase + FUD (50 °C); (**b**) DPEase + FUD (60 °C); (**c**) DPEase + FUD + GO (50 °C); (**d**) DPEase + FUD + GO (60 °C). The regions R1 and R2 marked with cyan color rectangles represent obvious variation in motion modes.

Cross-correlation maps for residues were computed by utilizing normal mode wizard plug-in module to expose impacts of immobilization of GO on the internal dynamics of related regions (Figure 11). The black and red represent the movement having strong positive correlation, while the light blue color represents the intensely anticorrelated motions. The diagonal region represented the motion correlation of the residue itself, so the diagonal part had the positive strongest correlation. The regions outside the diagonal described the motions of a residue relative to the other residues. The region R1 displayed the motion correlation of residues between loop β4-α4 and loop β5-α5, and the movement relativity between loop α1′-α1 and α8′ was represented by region R2. The region R3 yielded the strongly positive correlated movements in Model 1 (Figure 11a) and Model 3 (Figure 11c). However, the positive correlation of this region was greatly weakened in Model 2 (Figure 11b) and 4 (Figure 11d). This result was consistent with previous PCA and network results. The region R2 showed strong positive correlation movement except for Model 2. MD simulation at high temperatures weakened the positive correlation motions of residues in this region.

## 3. Discussion

Understanding the interaction mechanism of GO with DPEase at the GO-DPEase interface has great significance for promoting DTEase enzymes applications in bioproduction of D-psicose from D-fructose. Four initial models were constructed and performed 300 ns molecular dynamic simulations for each model in our work. Dedania et al. have immobilized *agtu*-DPEase on GO through experiments [29]. MD simulations showed that the *agtu*-DPEase can gradually be adsorbed onto GO surface over time. Similar phenomenon between proteins and nanoparticles have been observed in recent MD simulations. Different kinds of proteins could be adsorbed on different nanomaterials [36,37,38,39,40], which suggested that the MD simulation method can simulate the adsorption process of protein on GO, which provided a reliable guarantee for our further research. We found that several positively charged or hydrophilic residues were important anchors, inducing the adsorption of DPEase by interacting with GO. These residues had strong interactions with negative charged groups on the surface of GO.

The *agtu*-DPEase immobilized by GO (GO-*agtu*-DPEase) shows maximum activity at 60 °C [29]. By contrast, *agtu*-DPEase without GO is most active at 50 °C [41]. Experimental data has shown that the half-life of GO-*agtu*-DPEase enzyme at 60 °C was much higher than that of *agtu*-DPEase and other DPEase family enzyme [41,42,43,44]. Furthermore, the half-life of GO-*agtu*-DPEase enzyme was also higher than that of other immobilization materials [33,45]. Conformation of active sites are important factors affecting the specificity and efficiency of enzymes. We observed that two active site residues of *agtu*-DPEase were far away from the catalytic center when the simulations performed at 60 °C; however, there were no significant changes in the conformation of active site residues for GO-*agtu*-DPEase under the same temperature condition. To further investigate the underlying mechanism of GO enhancing the thermal stability of DPEase, we found that the DPEase only simulated at high temperature (60 °C) underwent significant conformational changes, especially in the loop α1′-α1, loop β4-α4, and α8′, compared to that performed at 50 °C. These findings were consistent with the crystallographic study [35]. It is noteworthy that the reasons for the structural changes in Model 2 were differ from Models 3 and 4. The dramatic structural changes in Model 2 was due to the stability of DPEase was destroyed at high temperatures. By comparison, Models 3 and 4 were due to the proteins successfully adsorbed on the surface of GO, resulting in inevitable changes in protein structure.

W14 in loop α1′-α1 shifted the positions obviously when binding substrate, so it is an important catalytic residue [35]. Our simulations showed that α8′ adsorbed on GO could prevent the loop α1′-α1 region from deviating the active center through hydrogen bond interaction. Crystallographic study manifested that W112 in the β4-α4 loop plays an important role in closing the active center and has obvious conformational changes. W112 is stabilized by a strong hydrogen bond forming with E156 in loop β5-α5 [35]. We observed that part of the β4-α4 loop region can be adsorbed on the surface of GO. Moreover, we found that there was motion correlation between loop β4-α4 and loop β5-α5 by analyzing the trajectory of MD simulations. The interactions between two loops helped W112 keep stable at high temperature. Interestingly, although DPEase can be adsorbed on GO at 50 °C, the loop β5-α5 moves away from the active center, so the enzyme activity was affected. The immobilization nanomaterial can protect enzymes from denaturation at high temperature [46]. In the meantime, immobilization could enhance enzyme rigidity and reduce conformation flexibility [47]. When the GO-*agtu*-DPEase enzyme was at 50 °C, the protein itself did not fluctuate greatly. Strong adsorption of GO led to excessive rigidity of protein, which will promote the loop to move in the opposite direction of GO and far away from the active center. So, it was not conducive to the occurrence of catalytic reaction. This could help explain why the GO-*agtu*-DPEase enzyme has the highest activity at 60 °C, not at 50 °C.

MD simulation is conducive to explain the interaction mechanisms between proteins and nanostructured materials when it is related to experimental data [12,48,49,50,51]. So, new insights could be provided for the comprehension of experimental results from the perspective of the dynamic behavior at atomic level. In case of our work, the above analysis results were consistent with the experimental data of enzyme activity [29]. So, MD simulations not only indicated that GO could enhance the thermal stability of DPEase, but also theoretically elaborated the mechanism of how GO interacted with DPEase and enhanced its thermal stability.

## 4. Methods

The initial coordinates of *agtu*-DPEase complexed with manganese ion and the frucose (FUD) were obtained from the RCSB Protein Data Bank (https://www.rcsb.org/, accessed on 3 July 2006) (PDB code: 2HK1) [35]. This crystal structure contains ligand (D-Fructose), so can be directly used for MD simulations. A nanostructure model of GO with the dimensions of 10 × 11 nm^2^ was prepared from the website (https://jerkwin.github.io/gmxtool/model/graphene.html, accessed on 5 April 2020), which is an online tool created by Jicun Li. The structure created with this tool has been optimized using xTB. The ratio of carbon to oxygen in GO is 4:1; this proportion of GO has been experimentally obtained [52]. Epoxy and hydroxyl groups were randomly attached to the carbon atoms of graphene basal plane.

The initial distance between the GO surface and the center of mass (COM) of the DPEase was 4.0 nm. The DPEase-FUD complex was positioned at the center, and the direction was randomly selected. To explore the dynamic characteristics of the DPEase-GO interaction, 300 ns MD simulations applying explicit water model were carried out for four systems. Four starting structures for MD simulations were prepared. Model 1: DPEase + FUD (50 °C), Model 2: DPEase + FUD (60 °C), Model 3: DPEase + FUD + GO (50 °C), Model 4: DPEase + FUD + GO (60 °C). Each system contains ligand and manganese ion. Four MD simulations were performed by the GROMACS 2018.3 [53] MD software package with Gromos54a7 force field [54,55,56]. The topology files of the GO generated by x2top suite embedded in the GROMACS [53] software package. GO sheet and the complex were embedded into the periodic boundary simulation box with SPC water molecules [57]. The size of the box was 10 × 11 × 9 nm^3^. The simulations of Model 1 and 3 were carried out at 50 °C, and Models 2 and 4 were simulated at 60 °C. Additionally, 100 ps pre-equilibrated MD simulations were performed in the NVT ensemble at 50/60 °C using a Berendsen thermostat [58], and the NPT ensemble was used for four systems where the Berendsen thermostat and the Parrinello–Rahman barostat [59] were applied, respectively, to maintain the pressure at 1 bar and the temperature at 50/60 °C. Four systems were performed in an NPT ensemble. The time steps of all simulations were set to 2 femtoseconds (fs), and the snapshots were recorded every 10 picoseconds (ps), setting the cutoff radius for the van der Waals interaction as 1.0 nm. The trajectory analysis were performed with GROMACS suite of programs [53] and VMD [60].

## 5. Conclusions

In this study, we performed MD simulations to elucidate how GO stabilized the DPEase at high temperature. Our results indicated that DPEase can be adsorbed onto the surfaces of GO surface through its cationic residues and hydrophilic residues. The active site of DPEase was stabilized at high temperature in the presence of GO, which was beneficial to maintain the catalytic activity of the enzyme. Our simulation results corroborated experimental observations and offered molecular insights into the interactions between DPEase and GO. The mechanistic understanding gained from this study should be useful for the design and development of DPEase.

## Figures and Tables

**Table 1:** Average backbone atoms RMSD of loop α1′-α, loop β4-α4, loop β5-α5, α8′, and α5 throughout 300 ns simulations in Å.

Regions	DPEase + FUD		DPEase + FUD + GO	
	50 °C (Model 1)	60 °C (Model 2)	50 °C (Model 3)	60 °C (Model 4)
loop α1′-α1	0.64 ± 0.17	1.89 ± 0.29	1.34 ± 0.24	1.47 ± 0.18
loop β4-α4	0.85 ± 0.35	1.55 ± 0.33	1.66 ± 0.34	1.59 ± 0.24
loop β5-α5	0.50 ± 0.077	0.52 ± 0.09	0.59 ± 0.09	0.60 ± 0.08
α8′	0.52 ± 0.22	1.71 ± 0.55	1.64 ± 0.53	1.85 ± 0.58
α5	0.74 ± 0.17	0.88 ± 0.30	1.01 ± 0.20	0.84 ± 0.17

**Table 2:** Hydrogen bond occupancies between protein (DPEase) and ligand (FUD) for four systems during 300 ns MD simulations.

System	Donor	Receptor	Occupancy (%)	Donor	Receptor	Occupancy (%)
DPEase + FUD (50 °C)	FUD:O1	E150:OE2	67.06	FUD:O6	E34:OE2	24.35
	FUD:O4	E244:OE2	64.52	R215:NH2	FUD:O1	19.13
	FUD:O1	E156:OE2	46.00	FUD:O6	E34:OE1	54.90
	FUD:O1	E156:OE1	45.16	H186:NE2	FUD:O1	34.88
	FUD:O5	E244:OE2	33.98	FUD:O3	E244:OE2	14.76
DPEase + FUD (60 °C)	FUD:O6	E34:OE1	40.77	FUD:O3	E244:OE2	32.21
	E34:OE1	FUD:O6	40.77	Y7:OH	FUD:O6	30.88
	FUD:O6	E34:OE2	39.91	H186:NE2	FUD:O1	29.28
	FUD:O1	E150:OE2	39.34	FUD:O4	E244:OE1	24.12
	FUD:O4	E244:OE2	39.14	FUD:O1	E156:OE2	10.12
DPEase + FUD + GO (50 °C)	FUD:O4	E244:OE2	60.99	FUD:O1	E156:CD	10.83
	FUD:O3	E244:OE2	62.43	FUD:O5	Y6:OH	11.41
	FUD:O6	E34:OE2	52.02	FUD:O6	E34:OE1	29.27
	FUD:O1	E156:OE1	17.54	R215:NH2	FUD:O1	6.12
	Y6:OH	FUD:O6	11.29	R215:NH1	FUD:O1	2.55
DPEase + FUD + GO (60 °C)	FUD:O4	E244:OE2	85.88	FUD:O1	E156:OE1	33.61
	FUD:O3	E244:OE2	61.38	Y6:OH	FUD:O3	21.65
	FUD:O6	E34:OE2	47.20	R215:NH2	FUD:O1	21.47
	FUD:O1	E156:OE2	38.68	Y7:OH	FUD:O6	14.27
	FUD:O6	E34:OE1	37.99	H186:NE2	FUD:O2	15.46

## Data Availability

Not applicable.

## Supplementary Materials

The following are available online at https://www.mdpi.com/article/10.3390/ijms221910813/s1.
