# Supplementary figures and images for: A Possible Mechanism of Graphene Oxide to Enhance Thermostability of D-Psicose 3-Epimerase Revealed by Molecular Dynamics Simulations
Source: Int J Mol Sci. 2021 Oct 6;22(19):10813. doi: 10.3390/ijms221910813 (PMC8509277; doi:10.3390/ijms221910813)

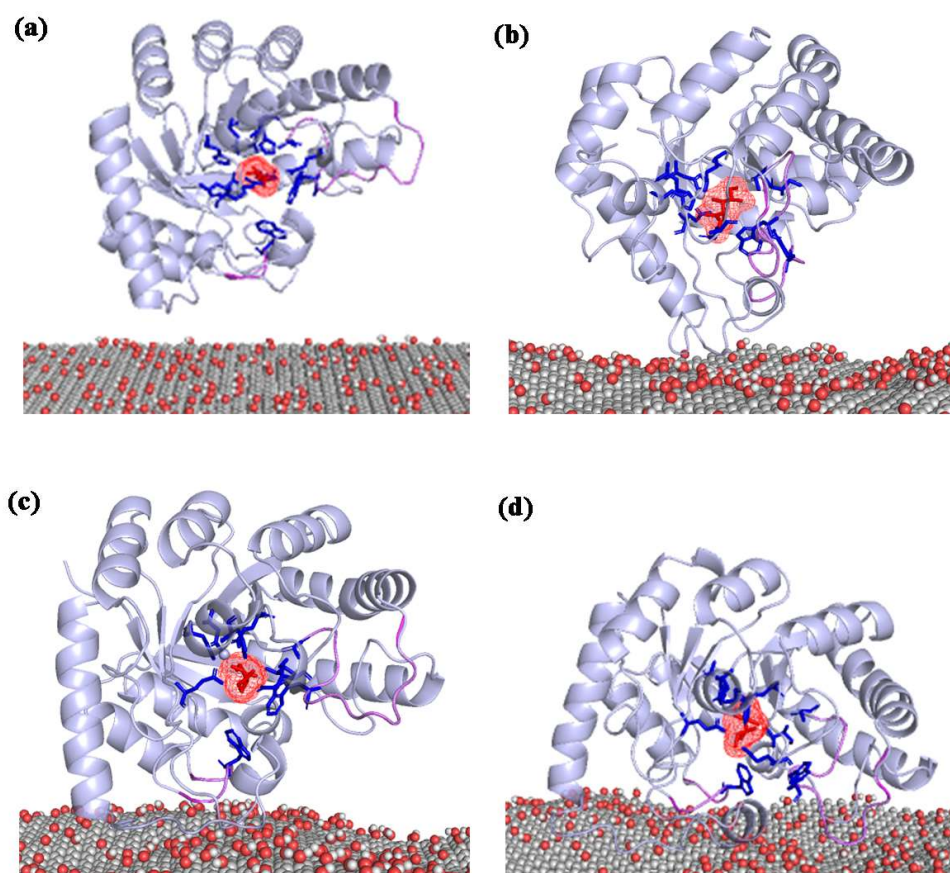

**Figure S1.** Adsorption process of enzyme on GO at 50 °C. The snapshots of (a) 0 ns; (b) 30 ns; (c) 70 ns; (d) 100 ns.

Supplement: Supplementary file 1 [file ijms-22-10813-s001.zip › ijms-1374565-supplementary.pdf]
